# Sustaining implementation facilitation: a model for facilitator resilience

**DOI:** 10.1186/s43058-021-00171-4

**Published:** 2021-06-21

**Authors:** Tanya T. Olmos-Ochoa, David A. Ganz, Jenny M. Barnard, Lauren Penney, Erin P. Finley, Alison B. Hamilton, Neetu Chawla

**Affiliations:** 1 HSR&D Center for the Study of Healthcare Innovation, Implementation and Policy (CSHIIP), VA Greater Los Angeles Healthcare System – Sepulveda, 16111 Plummer Street (152), North Hills, CA 91343 USA; 2 David Geffen School of Medicine, University of California at Los Angeles, Los Angeles, CA USA; 3 Veterans Evidence-based Research Dissemination and Implementation Center (VERDICT), South Texas Veterans Health Care System, San Antonio, TX USA; 4 University of Texas Health at San Antonio, San Antonio, TX USA; 5 Department of Psychiatry and Biobehavioral Sciences, David Geffen School of Medicine, University of California at Los Angeles, Los Angeles, CA USA

**Keywords:** Implementation facilitation, Workforce support, Quality improvement, Care coordination, Primary care

## Abstract

**Background:**

Implementation facilitators enable healthcare staff to effectively implement change, yet little is known about their affective (e.g., emotional, mental, physical) experiences of facilitation. We propose an expansion to the Integrated Promoting Action on Research in Health Services (i-PARIHS) framework that introduces *facilitation intensity* and *facilitator resilience* to better assess facilitators’ affective experiences.

**Methods:**

We used an instrumental case study and facilitator data (logged reflections and debrief session notes) from the Coordination Toolkit and Coaching initiative to conceptualize *facilitation intensity* and *facilitator resilience* and to better understand the psychological impact of the facilitation process on facilitator effectiveness and implementation success.

**Results:**

We define *facilitation intensity* as both the quantitative and/or qualitative measure of the volume of tasks and activities needed to engage and motivate recipients in implementation, and the psychological impact on the facilitator of conducting facilitation tasks and activities. We define *facilitator resilience* as the ability to cope with and adapt to the complexities of facilitation in order to effectively engage and motivate staff, while nurturing and sustaining hope, self-efficacy, and adaptive coping behaviors in oneself.

**Conclusions:**

Facilitators’ affective experience may help to identify potential relationships between the facilitation factors we propose (facilitation intensity and facilitator resilience). Future studies should test ways of reliably measuring *facilitation intensity* and *facilitator resilience* and specify their relationships in greater detail. By supporting facilitator resilience, healthcare delivery systems may help sustain the skilled facilitator workforce necessary for continued practice improvement.

**Trial registration:**

The project was registered with ClinicalTrials.gov (NCT03063294) on February 24, 2017.

Contributions to the literature

- Implementation facilitation is a widely used, dynamic, and challenging evidence-based strategy to support the implementation of change and improvement processes.
- As the evidence base for implementation facilitation effectiveness continues to grow and as healthcare delivery systems increasingly hire facilitators, additional research is needed that focuses on the facilitator’s perspective and the facilitator’s experience.
- To remain effective, facilitator resilience needs to be supported to reduce facilitator burnout, enhance facilitator effectiveness, and sustain the facilitator workforce.

## Background

Implementation science guides the systematic and effective uptake of evidence-based innovations into routine clinical practice [1]. Implementation facilitators are individuals trained to enable recipients of an innovation to effectively implement change in multiple settings [2]. Facilitators can be internal or external to the organization and often support activities like task management, accountability checks, process monitoring, and relationship building [2, 3]. Facilitators are well-suited to address a broad set of implementation challenges, e.g., resistance to change at all levels of the healthcare system [4, 5], constrained resources and limited implementation expertise [6], misaligned incentives and competing priorities [7], and diminished staff morale and burnout [8]. Facilitators have also been effective in improving the adoption of evidence-based guidelines in primary care settings [9–12], and in supporting quality improvement (QI) [13–15].

**Fig. 1:**
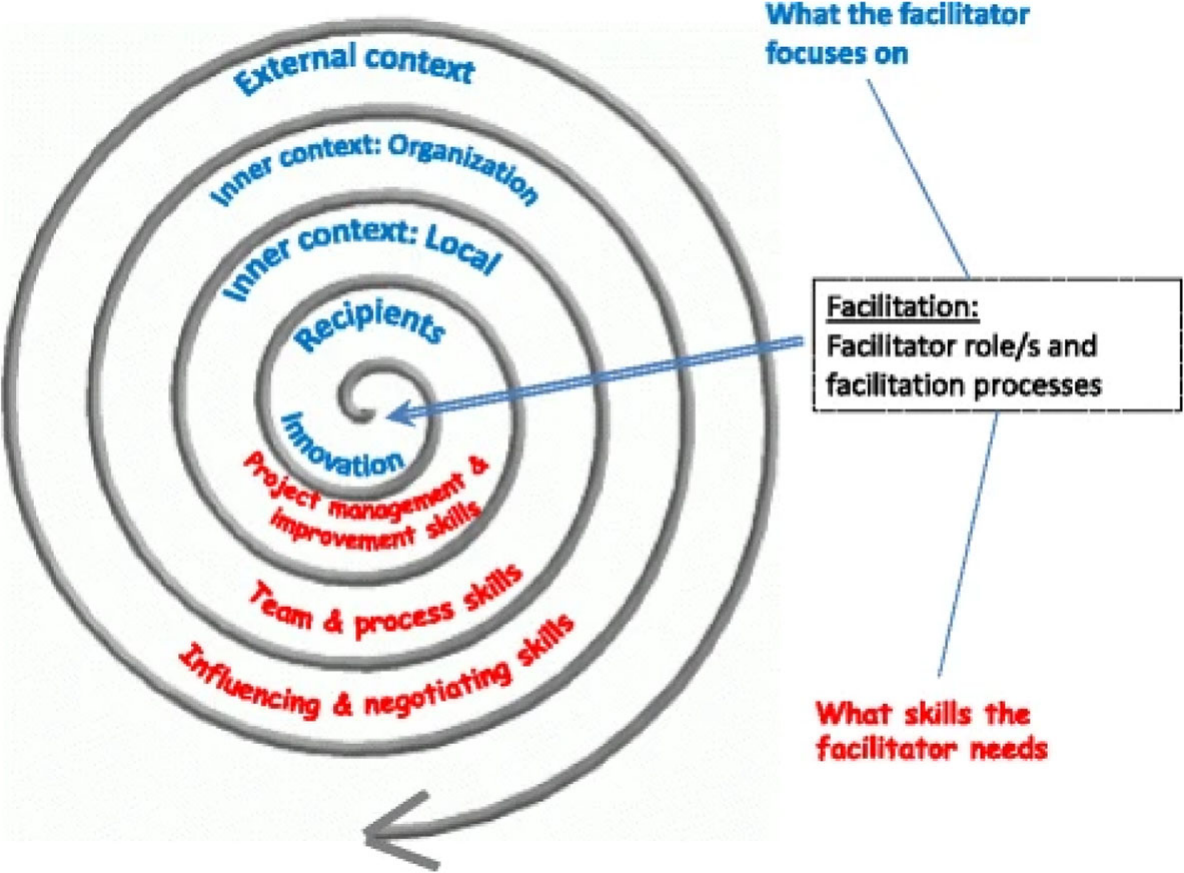
i-PARIHS framework (no changes made to original). Harvey G, Kitson A. PARIHS revisited: from heuristic to integrated framework for the successful implementation of knowledge into practice. *Implementation Science*, 2016. 11:33. http://creativecommons.org/licenses/by/4.0/ [16]

The Integrated Promoting Action on Research in Health Services (i-PARIHS) implementation framework, the most commonly used theoretical framework for understanding facilitation in implementation science [16], defines *facilitation* as the role and strategies used to enable implementation and the “active ingredient” that brings together an innovation, its recipients, and context to achieve successful implementation (see Fig. 1) [16]. Whether internal or external, facilitation involves collaborating with staff toward an implementation goal in complex and challenging healthcare environments, making it primarily a *relational* strategy. Additionally, facilitation that is longer in duration and frequency is associated with better implementation outcomes compared to less intense facilitation [11]. As such, effective facilitation can be affectively (e.g., emotionally, mentally, physically) demanding. Although facilitators’ perspectives are receiving more attention [10, 13, 17, 18], little is currently known about the affective impact of facilitation on the facilitator and how facilitators’ experiences may affect facilitator effectiveness, implementation outcomes, and ultimately sustainability of a facilitator workforce within healthcare delivery systems. In this paper, we review the i-PARIHS framework, present a case study that illustrates two distinct elements of facilitation (facilitation intensity and facilitator resilience) that have not been fully specified in previous studies, and propose an expanded conceptual framework that better reflects facilitators’ affective experiences.

## Methods

### The Coordination Toolkit and Coaching (CTAC) project: a facilitator case study

Case studies allow researchers to understand phenomena in depth and in their real-life, everyday contexts [19–21]. We use an instrumental case study design, which focuses more on the *issue* being researched than on the *case* from which the issue is analyzed [20, 22], to qualitatively explore facilitators’ experiences and specify them more fully in i-PARIHS. The *issue* we explore is the relationship between facilitation and facilitator experience and its potential impact on achieving facilitator effectiveness and implementation success. The *case* is the Coordination Toolkit and Coaching (CTAC) project.

The CTAC project is a QI initiative funded by the Veterans Health Administration (VA) to improve patients’ experience of care coordination between outpatient settings within and outside the VA [23], at sites confronting a variety of care coordination barriers [24]. Using a cluster-randomized design, matched pairs of VA clinics were recruited over time and randomized to either a passive strategy (access to an online toolkit) or an active strategy (distance-based facilitation plus online toolkit access) [23]. Two novice facilitators facilitated six VA clinic teams over a 12-month period; each facilitator was assigned three clinics. The two facilitators were both women VA employees with doctoral training in health services. Each facilitator had more than 10 years of experience working as an embedded researcher in primary care settings similar to the clinical sites participating in CTAC, and working with clinical staff on research and quality improvement efforts. Facilitators organized and hosted weekly facilitation calls for each of their assigned clinics (269 calls in total), answered project-related questions between scheduled calls, provided feedback on products generated by the teams (e.g., patient education brochures), and supported learning and practice of QI methodologies (e.g., usability testing), and data collection and analysis. Facilitators responded to project-related concerns from clinic teams via e-mail, phone, and instant messaging. Significant facilitator availability and flexibility were built into CTAC to encourage team engagement and project completion.

In CTAC, facilitators had responsibilities both to the funder (e.g., maintaining the overall project timeline) and to each clinic. In contrast to traditional research, the flexibility (e.g., sites’ selection of their own project topics) and inherent collaboration of CTAC facilitation helped to generate staff buy-in for the project through co-production of a project action plan, timeline, and products (e.g., workflow maps, clinic brochures). In addition, each facilitator had protected time to reflect on and adapt to feedback from the site-level implementation teams, from the larger CTAC team, and from the other facilitator to enhance facilitation effectiveness. Protected time to reflect and debrief accounted for 15–20% of the total facilitation effort, which was 20 h per facilitator, per week.

After each facilitation call, the facilitators logged reflections [25] in a database to document details about facilitation encounters (e.g., duration, modality, names of participants, open-ended summary) and to describe facilitation challenges and successes. Facilitators also debriefed in person or by phone after each call and kept debrief session notes. The CTAC team (principal investigator, project director, and two facilitators) met weekly to discuss facilitation progress, brainstorm QI strategies, review sites’ products and deliverables, solicit clinical advice from the principal investigator, elicit content expertise external to the CTAC team, and debrief about facilitation successes and challenges.

Discussions during the weekly CTAC team meetings prompted the team to begin thinking more deeply about the emotional demands of facilitation on the facilitators, and how facilitator experiences of those demands might impact the effectiveness of the facilitation strategy overall, and thereby the implementation outcomes. To inform our observations and interpretations, we conducted a targeted review of the facilitation literature by searching PubMed and Google Scholar for the terms “facilitation” and “facilitator,” combined with the terms “wellbeing/well-being,” “intensity,” “challenges,” “barriers,” “experience,” and “perspective.” We expanded our search further by reviewing the bibliographies of articles that met our search criteria and found gaps regarding facilitator experiences, particularly the *intensity* of the facilitation experience and the *resilience* necessary to persevere in the role. This thought exercise led us to perform a more detailed review of the templated reflections (*n* = 269) and debrief notes, using them as historical insight into the facilitators’ process and experience of facilitation. Using rapid qualitative analysis principles [26, 27], the two facilitators conducted an independent review of their logged reflections and debrief session notes to identify occurrences of positive demands (e.g., meeting more often than planned with highly motivated clinic sites) and negative demands (e.g., managing challenging team dynamics) on the facilitator. This review resulted in the identification of preliminary themes related to facilitation intensity and facilitator resilience. Of note, the focus of this review was to inform our reconceptualization of the i-PARIHS framework and not to generate empirical support for the preliminary themes identified through the review. These themes were then discussed between the facilitators and with the broader CTAC team to reach consensus on the challenges facilitators experienced, the strategies they employed to overcome those challenges, the perceived impact those challenges had on their effectiveness, and the activities and supports that bolstered facilitator morale. Consensus between facilitators was reached when they both agreed on what constituted a demand, and on the impact of that demand on the facilitator and the facilitation process. The CTAC team served as mediator when the facilitators had trouble reaching consensus. All members of the CTAC team reviewed and agreed on the definition of facilitation intensity and facilitator resilience.

## Results

### Facilitation intensity

During CTAC, we learned that different degrees of facilitator effort, or *facilitation intensity*, are needed to enable staff to implement change. We define facilitation intensity as “a quantitative and/or qualitative measure of both the facilitation tasks and activities needed to engage and motivate implementation, and the psychological impact on the facilitator of delivering the facilitation tasks and activities” [28]. In CTAC, facilitator intensity was assessed quantitatively by the frequency and duration of facilitation encounters (dose) (Olmos-Ochoa TT et al., submitted for publication) and qualitatively in our review of the written facilitator reflections. At all phases of implementation (pre, during, post), facilitators experienced multiple challenges and successes, and a spectrum of positive and negative emotions, resulting from facilitation encounters that were more or less immediately successful. During pre-implementation, for example, engaged CTAC teams whose expectations needed to be managed and whose desires for change transformed into actionable project goals were often perceived by the facilitators as more favorable to work with than teams that were less engaged and needed to be heavily supported in identifying project goals. Yet, both types of teams were viewed as equally fatiguing, since the energy expended by the facilitator to rein in one site and motivate the other were comparable. Similarly, while facilitation that was less successful could be taxing and disappointing, successful facilitation could also deplete the facilitator’s energy by being time-absorbing and intense. During implementation, the process of instructing staff on how and which data to collect in the course of usability testing required greater facilitation intensity than, for example, supporting staff to create a patient education brochure. Similarly, a direct confrontation with a clinic team member that lasted mere minutes could contribute more readily to facilitator fatigue and inefficacy than a longer encounter without conflict. In post-implementation, motivating teams to remain engaged with their planned sustainability efforts despite the conclusion of the formal facilitation strategy was challenging and weighed on the facilitators’ sense of commitment to the teams.

The intensity with which CTAC facilitators experienced the facilitation process within the same implementation effort varied not only from encounter to encounter, but also from facilitator to facilitator. A task or challenge that elicited a strong emotional response in one facilitator sometimes went emotionally unacknowledged by the other. This variability in facilitation intensity may be explained, in part, by the facilitator’s typical “emotional reactivity” to events, or their affective intensity—an individual characteristic that determines the strength of the emotional response to events and that can vary across individuals [29]. One example of this variation in emotional reactivity was visible in the facilitators’ reactions to clinic sites canceling facilitation calls. One CTAC facilitator internalized cancelations as a reflection of her facilitation and felt disorganized and uncertain about her effectiveness. In contrast, the other facilitator viewed cancelations as work to be made up later in the project period and assimilated the change with minimal reactivity. Additionally, debrief sessions were a venue for the supporting facilitator to provide the primary facilitator with her own (often less affectively intense) assessment of the same events. This allowed the primary facilitator to compare her own experience of the event with that of the supporting facilitator’s, brainstorm changes to her facilitation or to her reactivity, and adapt her facilitation process if necessary. CTAC facilitators alternated playing these supporting roles for each other throughout the project period.

### Facilitator resilience

One way in which facilitators may mitigate the effects of high-intensity facilitation is to cultivate resilience—the coping process that allows individuals to adapt and function effectively despite work-related challenges [30–32]. Facilitator resilience was previously defined by Kitson and Harvey as “not afraid of challenge/conflict; willing to engage in own professional development” [33]. For example, in one instance, a CTAC clinic selected a QI project related to chronic disease management. As a non-clinician, the facilitator took on the challenge by seeking out content and clinical experts to help develop strategies to support the clinic in its chosen project. Yet, the CTAC facilitators’ experience of resilience-building was more complex than this example indicates, varying by clinic and with the facilitation intensity required in each encounter over time.

Facilitators’ ability to cope with the fluctuating intensity of the facilitation process and the need to self-regulate outward displays of emotion to encourage recipient engagement and promote successful implementation were vital to facilitator resilience. We borrow from the sociology of work and nursing literature to more fully specify *facilitator resilience* and define it as the facilitator’s ability to cope and adapt to the complexities of facilitation (including facilitation intensity and emotional labor) to effectively engage and motivate recipients in implementation, while nurturing and sustaining hope, self-efficacy, and adaptive coping behaviors in themselves. “Emotional labor” was first coined by Hochschild (1983) to describe the effort expended by individuals to present a public display of appropriate emotions at work [34], defined as “the process of regulating both feelings and expressions for the organizational goals” through surface and deep acting [35]. Surface acting refers to the management of “observable expressions” [35–38], such as when CTAC facilitators needed to keep an even and upbeat tone to their voice on calls so that clinic teams did not pick up on the facilitators’ frustration. Deep acting refers to the managing of feelings and thoughts to produce an appropriate emotional reaction [35–38], such as when CTAC facilitators kept themselves from reacting negatively to emotional outbursts or confrontations from clinic members by justifying the members’ reactions with something external—“they are understaffed at the clinic today, so he must just be stressed.”

For nurses and other healthcare professionals, their personal and clinical (professional) skills are essential to the delivery of care, and as such, “their capacity to cope with their work cannot be separated from the content of the work itself” [38]. Similarly, facilitators use their personal and professional skills to deliver support to implementation teams. In CTAC, for example, facilitators listened sympathetically to teams as they vented their frustrations with leadership or with clinic processes, while actively seeking clues in the conversation to identify potential barriers to implementation success. Thus, facilitators’ ability to cope with and adapt to the complexity of facilitation cannot be separated from the facilitation process itself. As such, we postulate that the emotional labor facilitators expend during each encounter and across encounters during the facilitation period may accumulate and impact relational and implementation outcomes in the same manner that emotional labor by healthcare professionals can affect patient outcomes.

### Supporting greater facilitator effectiveness

In addition to illustrating our proposed inclusion of *facilitation intensity* and *facilitator resilience* as key components of facilitation, we also propose *facilitator effectiveness* as an interacting construct to assess how facilitators receive and process feedback about their effectiveness. Research on emotional intelligence in nurses demonstrated the importance of emotion self-repair—defined as the capacity of individuals to “interrupt their negative emotional states” by identifying, verbalizing, fostering, and otherwise extending their positive emotional states [39]. The repair process was shown to be positively associated with psychological wellbeing and quality of life. There were several instances when CTAC facilitators felt overwhelmed by the lack of progress made and/or by the challenging team dynamics on a call. During post-facilitation debriefs with the supporting facilitator, primary facilitators were able to freely discuss their frustrations without fear of judgment or reprisal from the clinic team, or of negative appraisal from supervisors. Providing the time and space for reassurance, constructive criticism, and emotional support were key to supporting facilitators and providing a sense of psychological safety. Facilitators were thus able to reflect and adapt their facilitation to improve effectiveness through a series of feedback mechanisms built into CTAC, including structured and recurring debrief sessions, recurring meetings with the facilitation team, protected time for facilitator reflection, and the collection of short-term outcomes (see Table 1).

**Table 1:** Sources of facilitator effectiveness feedback with CTAC examples

Sources	CTAC examples	Details
**Formal/informal input from recipients**	• Prior to facilitation, we asked facilitation recipients to provide their expectations of the facilitator and the facilitation process using a questionnaire	• One-time questionnaire fielded by CTAC project manager at baseline
	• Facilitators informally asked recipients how they felt the process was going during facilitation calls	• Ongoing solicitation of feedback by facilitators during weekly calls
	• Recipients were asked to evaluate the facilitators’ efforts at the project midpoint and endpoint using formal qualitative interviews	• External evaluators conducted qualitative semi-structured interviews at 6 months and 12 months from the start of facilitation
**Structured and recurring debrief sessions**	• The two facilitators debriefed with each other after each weekly facilitation call	• Debrief sessions ranged from 15 to 60 min in length • 269 facilitation calls total across six clinic sites
	• Additional informal debrief sessions occurred during the recurring weekly meetings with the larger CTAC team	• CTAC facilitators provided weekly updates to the larger CTAC team (PI, project manager, evaluators), which included debriefing about facilitation challenges • Meetings were 60 min
**Recurring meetings with CTAC team**	• Weekly meetings that included the principal investigator of the project, the project manager, and the two facilitators, as well as other experts on an as-needed basis	• Meetings were ongoing from 2016 when the first site was enrolled in CTAC through 2020 when the project concluded • Meetings were 60 min
**Protected time for facilitator reflection**	• Facilitators were provided time to complete a templated reflection form after each facilitation call to document the content of the call and record facilitation challenges and successes	• Written reflections were completed for 269 calls and took < 5 min to complete • The templated form included prompts for the call’s date, duration, participants, open-ended summary of what transpired on the call, and descriptions of facilitation challenges and successes
	• Reflection also occurred informally during the structured debrief sessions	• Debrief sessions between the two CTAC facilitators often included verbal reflections about the facilitation process
**Collection of short-term process outcomes**	• Each clinic site team collected data throughout the 12-month project to track progress toward attainment of its implementation goals	• These data were project-specific, collected by the clinic site teams, and often included usability testing of patient and staff-facing products (e.g., brochures, workflow maps), tracking product distribution within the clinic, and auditing administrative processes (e.g., changes in the number of walk-in patients)

### Facilitator effectiveness and implementation success

**Fig. 2:**
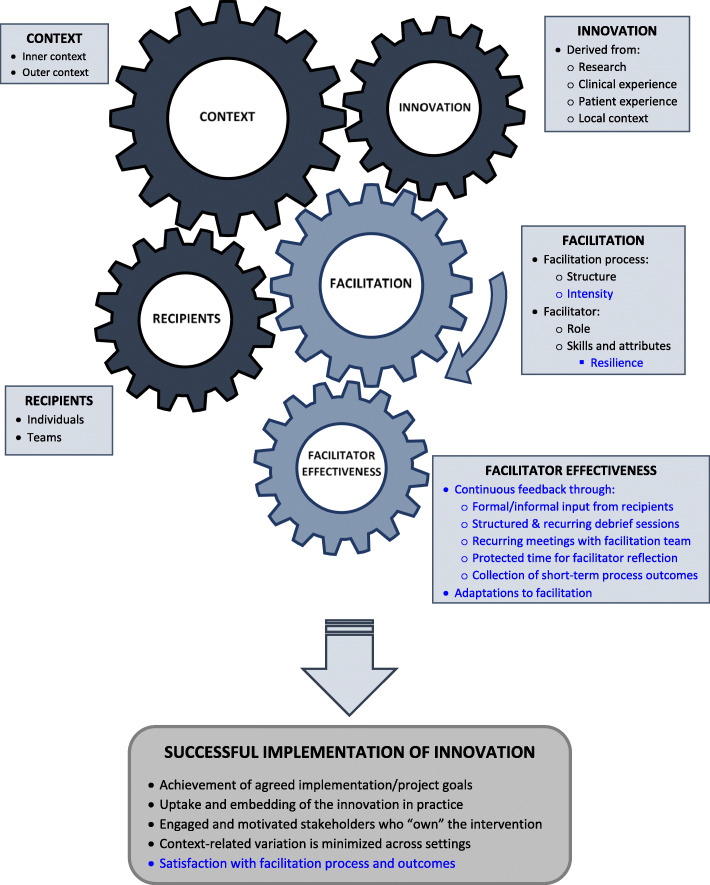
Expanded facilitation conceptual framework. Facilitators’ experience of facilitation, including their *facilitation intensity* and *facilitator resilience* may impact *facilitator effectiveness*. Supporting facilitator effectiveness through continuous feedback and adaptation may improve effectiveness and consequently, successful implementation

Figure 2 illustrates how an existing implementation framework (i-PARIHS) can be adapted to include the facilitation constructs we propose (*facilitation intensity* and *facilitator resilience*), as well as their potential relationship to *facilitation effectiveness* and implementation outcomes. We postulate that the fluctuations in facilitation intensity experienced by the facilitators during their facilitation process may impact their effectiveness if they are not properly supported. In turn, impacts on facilitator effectiveness may have important implications for implementation success. To account for the role of facilitator effectiveness in implementation success, we propose assessing stakeholders’ *satisfaction with the facilitation process and facilitation outcomes.* Stakeholders may include the recipients of facilitation and leadership at the implementation site, the facilitators themselves, and the research or facilitation team supporting the facilitators. Continuous feedback to the facilitators throughout the facilitation period can be beneficial at all stages of implementation. To what extent an innovation is integrated into the host organization and its subsystems (penetration) and maintained over time (sustainability) is an important marker of implementation success [40]. In late implementation stages, satisfaction with facilitation may provide valuable context for assessing broader implementation outcomes.

## Discussion

Using CTAC as a case study, we focused on *facilitation intensity* and *facilitator resilience* to better understand the facilitation experience and provide recommendations for how to augment resilience and *facilitator effectiveness*. CTAC facilitators experienced facilitation as a high-intensity and long-term process supported across six geographically and contextually different sites over a three-year period. The facilitators were external to the clinic sites and supported each site’s implementation team on a weekly basis over 12 months, often balancing the needs of multiple sites at the same time. CTAC facilitators used reflective writing and oral debriefs with each other to work through the affective impact of the facilitation process on their effectiveness and their wellbeing. The CTAC team, to whom facilitators reported their progress with each site, also provided additional feedback and support. Having protected time to reflect (verbally and in writing) on their facilitation process allowed CTAC facilitators to think critically about their facilitation, to make needed adaptations to their process, and to refine their facilitation to enhance their effectiveness. To support facilitators, we propose a continuous feedback process to provide facilitators with a variety of information and resources to cope with intensity, strengthen resilience, and understand, adapt, and improve their effectiveness. In CTAC, design elements of the program supported facilitators by providing multiple opportunities for facilitators to reflect, debrief, and brainstorm after all facilitation encounters, especially those that were challenging and high intensity. This allowed facilitators to step back emotionally and mentally from the facilitation process by engaging and relying on the feedback of others to process encounters.

As healthcare organizations hire, train, and rely more on implementation facilitators to support change efforts, understanding how best to support facilitator effectiveness and wellbeing, particularly in the challenging implementation contexts (e.g., during a global pandemic), is necessary for success and to sustain the facilitator workforce over time [41, 42]. To date, most facilitation research has focused on identifying and supporting the technical needs of facilitators through didactic training and on-the-job experience [17, 43, 44], with limited focus on the affective experience of facilitation, which we posit also influences facilitator effectiveness. In CTAC, the facilitators’ ability to respond and adapt to facilitation challenges (facilitator resilience) emerged as having potentially important implications for sustaining facilitator effectiveness and implementation success. As an active relational process, facilitation requires the confluence of a facilitator’s multiple technical skills and personal attributes to evaluate and respond in real time to the technical and relational needs of implementing staff working in highly complex healthcare settings [16, 45]. However, existing definitions of facilitation intensity (or dose), have primarily focused on the time (frequency and duration) facilitators spend conducting facilitation [11], and less on the variation in energy (mental, emotional, physical) expended by facilitators during each encounter and cumulatively across encounters. This paper further specifies the definition of facilitation intensity to include the affective impact on the facilitator of conducting facilitation tasks and activities over time [28].

Our findings suggest that for healthcare organizations to develop an effective infrastructure for facilitator support, it is necessary to build into the design of practice improvement efforts the ability for facilitators to reflect on and document their affective experience of the facilitation process. However, there are some limitations to the generalizability of our findings. Although CTAC was a QI initiative in VA primary care outpatient clinics, it differed from many locally initiated time- and resource-limited QI projects in that a rigorous evaluation of the initiative was expected (and therefore financially supported) by the funder. This requirement allowed us to build in the time and resources for reflection and to collect facilitator data, which may not be possible or supported in more resource-constrained environments or for internal facilitators who may be balancing their day-to-day responsibilities to the organization (e.g., care delivery, administration) with those of their facilitation. Nonetheless, the written reflections were brief, often taking CTAC facilitators between one to 5 min to complete, and may have the potential to be replicated in time-constrained settings and for internal facilitators balancing competing demands. An additional limitation on generalizability is that both CTAC facilitators had years of experience working in primary care clinical settings and with clinical staff, which allowed them to focus on the facilitation process itself rather than having to assemble primary care knowledge. Nonetheless, both facilitators were new to implementation facilitation, which may explain in part the need for the added and recurring supports (reflection and debrief activities) that more experienced facilitators may need less of or find unnecessary.

Facilitator data, particularly the reflections, were gathered in real time and reflect the facilitators’ thoughts and emotions proximal to the facilitation encounters. As such, the data focus on the facilitators’ affective experience and capture their thoughts about how to improve their effectiveness. Although these qualitative data cannot operationalize the theorized relationships between the facilitation factors we propose (facilitation intensity, facilitator resilience, and facilitator effectiveness), they do highlight potential areas for further research. Future research on facilitation should seek to operationalize the link between facilitator resilience and facilitator effectiveness, and to parse out the relationship between facilitator experience (novice to expert) and the facilitator’s ability to cope with facilitation intensity. Furthermore, researchers should develop measures that evaluate facilitation intensity beyond its frequency and duration to capture the energy expended by facilitators in relation to facilitator resilience. More qualitative insights are also necessary to understand how facilitators experience the facilitation process, including how they experience burnout and how to address it, and to identify the resources they may need to be supported and effective in their role. This focus on the supports for facilitation is important if facilitation and similar implementation strategies are to be sustained.

## Conclusions

Implementation facilitators are uniquely positioned to guide staff in implementation and process improvement. However, given the challenges of delivering facilitation, healthcare delivery systems that hire QI and implementation staff may benefit from understanding how to sustain facilitators’ efforts. Facilitators who can withstand the demands of facilitation and succeed in implementation acquire needed institutional, content, and facilitation knowledge. Supporting resilience in facilitators may help sustain the skilled facilitator workforce necessary for continued practice improvement.

## Data Availability

Consents associated with primary data collection for clinician/staff participants in CTAC did not include permission to share data in publicly available repositories. De-identified administrative datasets may be eligible for future data sharing once national VA guidance on request and distribution processes are provided (in process). Final datasets will be maintained locally until enterprise-level resources become available for long-term storage and access.

## Abbreviations

CTAC: Coordination Toolkit and Coaching
i-PARIHS: Integrated Promoting Action on Research in Health Services
QI: Quality improvement
VA: Veterans Health Administration
